# Giant Endoplasmic Reticulum vesicles (GERVs), a novel model membrane tool

**DOI:** 10.1038/s41598-020-59700-1

**Published:** 2020-02-20

**Authors:** Mona Grimmer, Kirsten Bacia

**Affiliations:** Biophysical Chemistry, Institute of Chemistry, Charles-Tanford Protein Center, University of Halle, Kurt-Mothes-Str. 3 A, 06120 Halle, Germany

**Keywords:** Membrane biophysics, Biological techniques

## Abstract

Artificial giant vesicles have proven highly useful as membrane models in a large variety of biophysical and biochemical studies. They feature accessibility for manipulation and detection, but lack the compositional complexity needed to reconstitute complicated cellular processes. For the plasma membrane (PM), this gap was bridged by the establishment of giant PM vesicles (GPMVs). These native membranes have facilitated studies of protein and lipid diffusion, protein interactions, electrophysiology, fluorescence analysis of lateral domain formation and protein and lipid partitioning as well as mechanical membrane properties and remodeling. The endoplasmic reticulum (ER) is key to a plethora of biological processes in any eukaryotic cell. However, its intracellular location and dynamic and intricate tubular morphology makes it experimentally even less accessible than the PM. A model membrane, which will allow the afore-mentioned types of studies on GPMVs to be performed on ER membranes outside the cell, is therefore genuinely needed. Here, we introduce the formation of giant ER vesicles, termed GERVs, as a new tool for biochemistry and biophysics. To obtain GERVs, we have isolated ER membranes from *Saccharomyces cerevisiae* and fused them by exploiting the atlastin-like fusion protein Sey1p. We demonstrate the production of GERVs and their utility for further studies.

## Introduction

Native biological membranes, especially the internal membranes in eukaryotic cells, constitute a complex and experimentally challenging object of study in cellular biophysics. For example, visualization by fluorescence microscopy techniques is particularly difficult for membranes that exhibit complex three-dimensional morphologies below optical resolution and a dynamic behavior like the ER network makes it a challenge to analyze the mobility of ER-resident proteins quantitatively^1,2^. Both the complex morphology and the internal location of the ER hamper also mechanical manipulation, as in patch clamp. It is also often difficult to deliver reagents, including purified proteins, to internal membranes for studying their influence on the membrane. Although the PM can be permeabilized with detergent to permit supplemented proteins to reach the ER^3,4^ this approach comes with potential side effects on internal membrane structure.

On the other hand, when membrane-related processes are reconstituted outside the cell using artificial model membranes to facilitate experimental access, massive simplifications ensue and critical choices need to be made. These concern for instance the lipid composition, limitations in the number of membrane protein(s) that can be handled by reconstitution, and, typically, a loss of bilayer asymmetry. Well-established artificial bilayer membrane models include supported lipid bilayers and vesicles of various sizes, including giant unilamellar vesicles (GUVs). GUVs feature a broad application spectrum: (i) They offer large, low curvature surfaces, e.g. for studying membrane fusion and fission events^5^. (ii) Their inner aqueous lumen is separated from the outer aqueous phase, allowing transport assays. (iii) They are reasonably stable. (iv) Membrane tension is tunable by osmotic tension or pipette aspiration. (v) Environmental conditions such as temperature and aqueous phase compositions can be manipulated. Furthermore, the quasi-planarity of the GUV membrane on the spatial scale of confocal microscopy permits quantitation of the lateral diffusion of fluorescent membrane components by fluorescence recovery after photobleaching and fluorescence correlation spectroscopy (FCS). Unfortunately, reconstitution of membrane proteins into GUVs to create proteo-GUVs^6^ is far more challenging than into small vesicles.

Giant vesicle production from native membrane material is a sought-after method, because these vesicles combine the best of two worlds: the native complexity and genetic controllability of the natural membrane with the simple geometry and accessibility of giant vesicles. So far, to our knowledge, only one such natural membrane model system, namely GPMVs, is well established. GPMVs are generally produced by chemically induced blebbing of the PM. In the case of erythrocyte ghosts, PM material has also been remodeled into giant vesicles by electroformation^7^. Over the years, GPMVs have proven a versatile model system for studying PM-related processes. GPMVs served to investigate lipid segregation into liquid-ordered and liquid-disordered phase^8^ and the distribution of lipids and membrane proteins into domains of different order^9,10^. Further GPMV studies analyzed e.g. protein translocation across the PM^11^, the path of cell penetrating peptides^12,13^, mechano-elastic membrane properties^14^ and membrane fusion processes, e.g. during HIV infection^15^.

Since the ER has a plethora of functions in the cell, it is highly desirable to devise a method for generating giant vesicles from ER membranes while preserving protein function. These GERVs are likely to facilitate and advance studies on co-translational membrane protein insertion, lipid synthesis, protein modifications, ER contact sites, unfolded protein response, ER-associated protein degradation, ion channels and calcium signaling as well as vesicular transport of newly synthesized proteins and lipids.

Purified ER membranes were previously remodeled into giant vesicles by electroformation^16^, which required ER membranes to be dried, dissolved in organic solvent and dried again. The treatment with organic solvent is prone to denaturing membrane proteins and breaks up native membrane architecture, limiting its range of applications. Alternatively, half-dried natural membranes and synthetic lipids were mixed to facilitate electroformation^17^.

**Figure 1:**
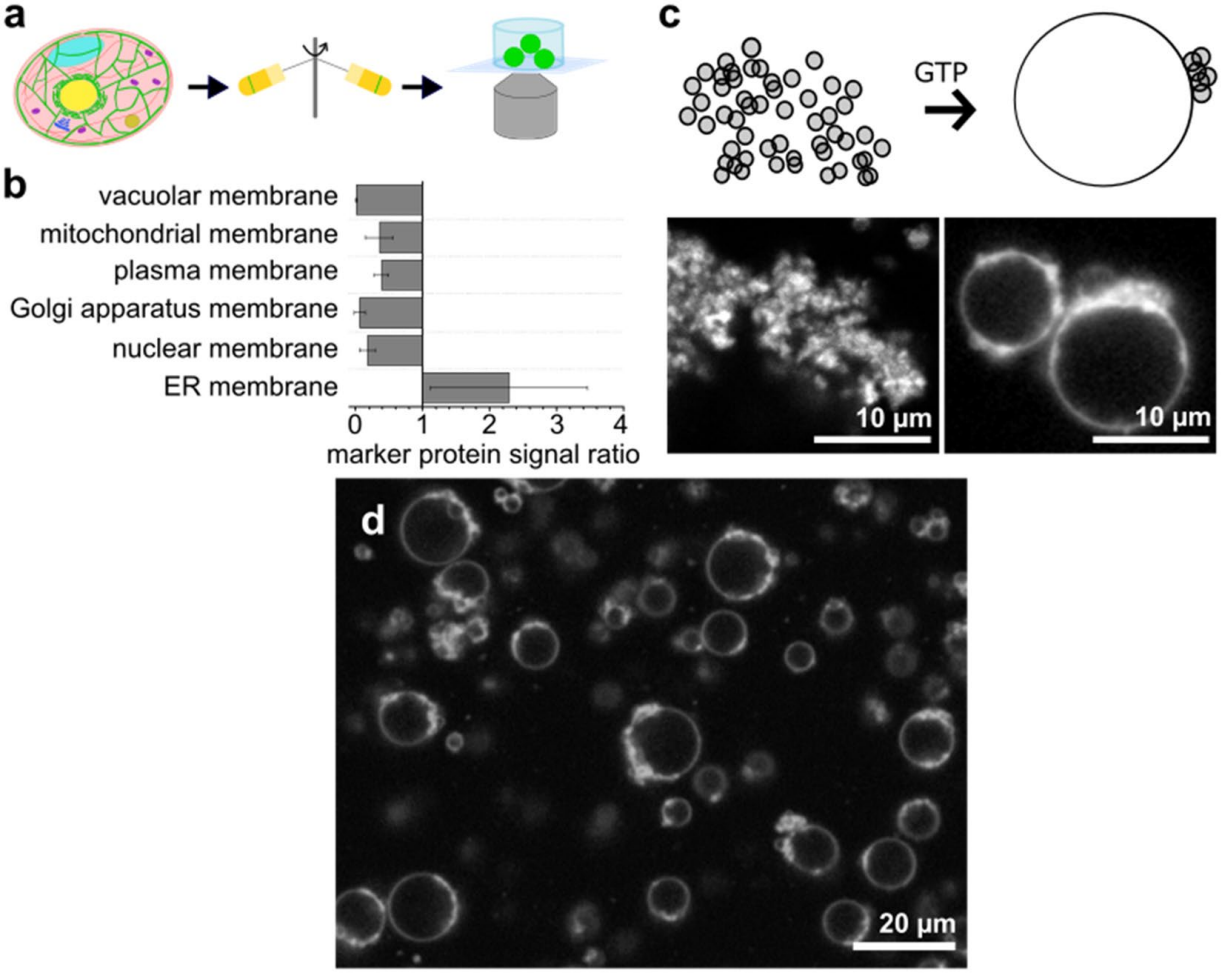
Giant ER vesicle formation (**a**) Schematic view. Sey1p-expressing cells are cultured and fractionated by multiple centrifugation steps to purify ER membranes. Sey1p-containing ER membranes are incubated with GTP to yield giant ER vesicles (GERVs), usable in confocal microscopy and other techniques. (**b**) Subcellular fractionation. Ratios of marker signals for different cellular organelles in the final relative to the starting material show enrichment of ER membrane and depletion of other organelle membranes. (**c**) GERV formation. Schematic view (top) and confocal fluorescence images (bottom) of GERV formation in the presence of 5 mM GTP. (**d**) Representative image of GERV formation with reconstituted Bet1p-mCherry. Membranes in (**c**) and (**d**) are fluorescently labeled with FM1-43.

Here, we present an alternative approach that keeps the membrane in its native state during giant vesicle formation (Fig. 1a). First, ER membranes are isolated from *S. cerevisiae* cells. Next, the purified ER membranes are fused to form giant vesicles by exploiting the atlastin-like ER-homeostasis protein Sey1p^18^. Reconstituted Sey1p is known to fuse liposomes^18,19^, but we noticed that with *S. cerevisiae* ER membranes, Sey1p overexpression was needed to produce ER vesicles of truly giant size, similar in size to the vesicles produced from *Xenopus laevis* extracts by Dreier & Rapoport^20^. This approach preserves the native lipids and membrane proteins of the ER, while remodeling its shape into the desired giant vesicles. As a proof-of-principle, we show that GERVs can be employed in confocal microscopy, immunostaining and FCS.

## Results and Discussion

### Purification and fusion of microsomes

*S. cerevisiae* cells that express Sey1p were grown and harvested. Cell walls were digested, the resulting spheroplasts disrupted and ER membranes purified by subcellular fractionation according to Wuestehube & Schekman (1992)^21^ with modifications. During the isolation process, the intricate ER membrane network fragments into ER-derived vesicles called microsomes. Marker proteins for the different organelles^22^ were used to optimize the preparation and show the successful purification of microsomes (Fig. 1b, Supplementary Fig. S1). The GTPase Sey1p is known to fuse opposing membranes by dimerization upon GTP binding^18,19^. Addition of GTP to isolated Sey1p-overexpressing ER membranes caused fusion and resulted in GERVs (Fig. 1c). GERVs were also formed when purified ER membranes were genetically enriched with Sey1p and a further ER-membrane protein (Bet1p-mCherry, Fig. 1d).

### Optimization of fusion conditions

**Figure 2:**
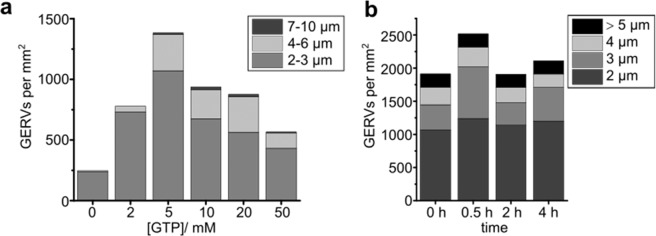
GERV formation and stability as a function of GTP concentration. (**a**) GERV formation. Number of GERVs for each diameter range were counted per area of coverslip. GERVs larger than 2 µm in diameter and with at least one third of their perimeter free of aggregates were considered adequate for further experiments and considered. Diameters were measured in micrometers and rounded to integers for classification. Incidence values are averages from four independent preparations of GERVs. From each of the four preparations, aliquots were taken and subjected to nucleotide concentrations between 0 to 50 mM GTP. In three out of the four microsome preparations, the 5 mM GTP condition yielded the largest density of GERVs. In one out of the four preparations, 10 mM GTP was most successful. (**b**) GERV stability. The number and size distribution of the GERVs were evaluated without apyrase (0 h) and 0.5 h, 2 h, and 4 h after addition of 0.01 U ml^−1^ apyrase. Quantification criteria were applied as described for (**a**). Values are averages from two apyrase assays.

To establish optimal fusion conditions for GERV formation, GTP concentration was varied. Fluorescently labeled microsomes were incubated with 0 to 50 mM GTP (Fig. 2). The highest yield of GERVs was obtained at 5 mM GTP. As small vesicles are indistinguishable from unfused microsomes, only vesicles larger than 2 µm were counted. Most GERVs had diameters up to 3 µm while some measured up to 10 µm in diameter (Fig. 2a). At higher GTP concentrations, the overall yield of GERVs declined. Hence, all further experiments were carried out at 5 mM GTP.

Next, we tested if, once formed, GERVs are stable in the absence of GTP. To this end, the enzyme apyrase, which breaks down GTP to GMP and phosphate (Supplementary Fig. S2), was added to preformed GERVs. Stability was monitored by assessing the number and sizes of GERVs at different time points (Fig. 2b). Even 4 hours after apyrase addition, there is still a considerable number of GERVs of all the sizes. Hence, after formation, excess GTP is no longer necessary to maintain GERV structure. The quality of the GERV lipid bilayer membranes was routinely judged by visual inspection of confocal overview images (Fig. 1d, Fig. S4a). It can be confirmed by quantitative image analysis (Fig. S4c). Areas with adhering aggregates are clearly distinguished from areas consisting of uniform bilayer membranes.

### Proof-of-principle: GERVs as a membrane model

**Figure 3:**
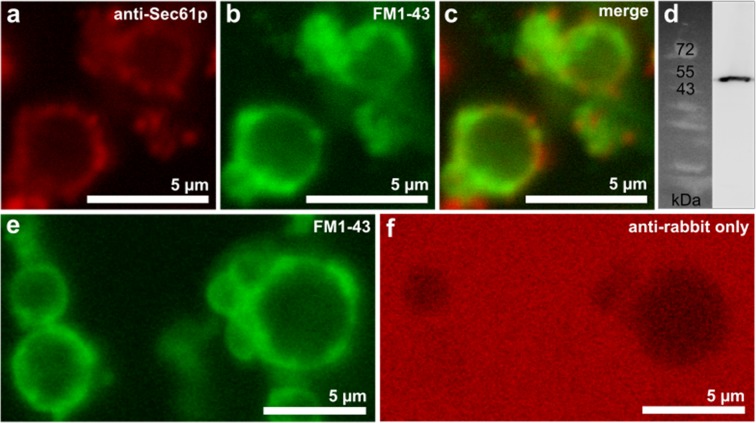
Immunostaining and confocal microscopy of the translocon Sec61p located in GERVs. (**a**) Sec61p was stained using anti-Sec61p (from rabbit) as the primary antibody and red fluorescent Alexa-647 labeled anti-rabbit as the secondary antibody. Antibodies were added sequentially to the GERVs (see SI). Panel  (**b**) shows the corresponding lipid bilayer labeling with FM1-43 (green) and (**c**) an overlay of both images. (**d**) Western blot of purified microsomes with anti-Sec61p anti-rabbit-HRP antibodies (see Fig. S1b for more lanes). Panels (**e**,**f**) display GERVs, which underwent the same immunostaining procedure as in (**a**–**c**), with the exception that the primary anti-Sec61p antibody was omitted.

To demonstrate the utility and versatility of the new, native model membrane, GERVs were employed in three types of fluorescence microscopy-based experiments. Firstly, using a classical immunostaining approach, the translocon, which translocates newly synthesized peptides into the ER, was visualized with a non-fluorescent primary (1°) anti-Sec61 antibody in combination with a red fluorescent secondary (2°) antibody. The Sec61 translocon colocalized with the green-labeled GERV bilayer (Fig. 3a–c). Binding specificity was checked in two control experiments: The single band at the molecular weight of Sec61p in the western blot showed that anti-Sec61p binding is specific to Sec61p (Fig. 3d). Omitting the 1° antibody from the immunostaining of the GERVs showed that the fluorescent 2° antibody was no longer enriched at the membrane, arguing against unspecific binding of the 2° antibody (Fig. 3e,f). The immunostaining experiment shows that GERV membranes contain Sec61p and could be used for studies of protein translocation.

Secondly, the feasibility of incorporating additional membrane proteins into GERVs by genetic means was explored. Unlike proteo-GUV preparations, GERV preparations do not require proteins to be purified and reconstituted, thereby reducing both labor and potential loss of protein structure and function. We chose two transmembrane proteins for constructing fluorescent fusions: the SNARE protein Bet1p, which is involved in trafficking between ER and Golgi^23,24^, and the guanine nucleotide-exchange factor, Sec12p, which localizes to the ER^25^. GERV-formation from microsomes of Bet1p-mCherry expressing cells (Fig. 1d) shows that the incorporation of an additional membrane-protein did not obstruct GERV formation.

**Figure 4:**
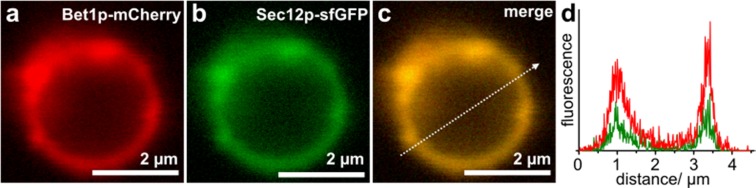
Confocal microscopy of GERVs containing fluorescent fusions of membrane proteins. (**a**–**c**) GERV formed by mixing microsomes from two Sey1p-cell lines, one expressing Bet1p-mCherry (shown in red), the other expressing Sec12p-sfGFP (shown in green). (**d**) The intensity plot along the indicated line shows the co-localization.

To study multiple membrane proteins of interest, GERVs offer a flexible way of incorporating multiple membrane proteins by exploiting the fusion between microsome populations (Fig. 4). Microsomes purified from Sey1p-cells expressing red-fluorescent Bet1p-mCherry and those from Sey1p-cells expressing green-fluorescent Sec12p-sfGFP were mixed and observed to fuse to each other in the presence of GTP, generating double-labeled GERVs, which contained both Bet1p-mCherry and Sec12p-sfGFP (Fig. 4).

Thirdly, we analyzed the diffusion of a transmembrane protein in GERVs by fluorescence correlation spectroscopy (FCS). FCS has been used for measuring the mobility of membrane components in many applications. It is readily performed on the PM, on GPMVs, on GUVs and on other model membranes^6,26–29^. FCS and its dual-color cross-correlation variant also allow analyzing binding of molecules to native and artificial membranes^29–31^. In contrast to the PM, the topology of the ER does not allow a straight-forward application of FCS and related techniques to quantitate molecular mobility and binding. We therefore tested if GERVs with their quasi-planar topology can serve for FCS.

**Figure 5:**
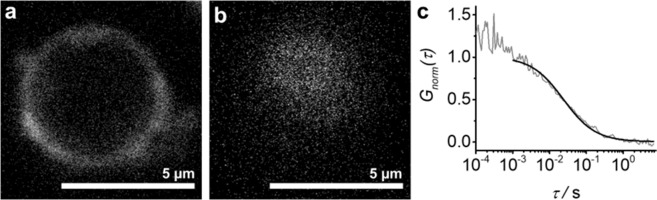
GERV containing a low density of Bet1p-sfGFP for FCS and a high density of Bet1-mCherry for imaging. (**a**) Confocal slice at the equator. (**b**) Confocal slice at the top, where the FCS focus is positioned. (**c**) Representative example of an FCS curve, measured at the top of a GERV, and the corresponding fit.

Since FCS requires a low density of fluorescent particles, whereas in confocal imaging higher concentrations are preferred, microsomes from Sey1p-cells expressing green fluorescent Bet1p-sfGFP and microsomes from Sey1p-cells expressing Bet1p-mCherry were mixed at a 1:   4000 ratio (based on total protein concentrations). This approach again highlights the flexibility bestowed by fusing microsomes from separate cellular preparations. Resulting GERVs were imaged in the red channel (Fig. 5a,b) and FCS measurements performed in the green sfGFP-channel (Fig. 5c), yielding a diffusion coefficient of *D* = (0.28 ± 0.1) µm^2^ s^−1^. The diffusion of the single span transmembrane protein Bet1p-sfGFP in the GERVs was approximately 9-fold slower compared to the diffusion of Alexa488-labeled Bet1p in artificial GUVs, prepared from ‘major-minor mix’^32^, a mixture of predominantly unsaturated lipids (Supplementary Fig. S3).

In GPMVs, Worch *et al*.^29^ found the diffusion of a single-span transmembrane protein to be slowed down by a factor of 5.5 compared to GUVs from unsaturated lipids. In analogy to GPMVs, the feasibility of FCS measurements on GERVs lays the foundation for comparative analyses of the diffusion of membrane components in GERVs and GUVs, including investigations of heterogeneity and compartmentalization. Moreover, recruitment and affinities of proteins interacting with the ER could be quantitated using this novel tool, whose complexity lies in between cells and artificial model membranes.

## Conclusion

GERVs are introduced as a new model membrane tool for investigating the plethora of processes connected to the ER membrane. Proof-of-principle applications involving fluorescent immunostaining as well as combinations of fluorescent membrane protein fusions are demonstrated. The membranes are amenable to confocal imaging as well as diffusion analysis by FCS. Further applications can be envisioned in analogy to and beyond GPMVs^33^. GERVs may be used to investigate enzyme functions linked to the ER membrane, lipid metabolism, amyloid fibril formation and effects of drugs. They may facilitate membrane protein production and serve as starting material for other model membranes, such as continuous supported membranes. In conclusion, we expect that this membrane tool will be highly useful in a large range of future studies in the field of membranes.

## Material and Methods

### Strains and vectors

The sequence of Sey1p was purchased from Dharmacon (Horizon Discovery) in a BG1805 vector. A stop codon was included between the Sey1p sequence and tag-sequences via polymerase chain reaction (PCR) with the following primers: 5′-GCAAAAAGAAAAA TGA AACCCAGCTTTCT and 5′AGAAAGCTGGGTT TCA TTTTTCTTTTT GC. Sec12p-sfGFP and Bet1p-sfGFP sequences were embedded into the GAL1-promotor controlled single copy vector pGAL413^34^. Bet1p-mCherry was embedded in pGAL415^34^. Cloning was performed using the conventional restriction site cloning procedure for Bet1p-sfGFP and the InFusion Cloning Kit (Takara) for Sec12p-sfGFP and Bet1p-mCherry. Used primers were: Bet1p-mCherry 5′-GGA TTC TAG AAC TAG TAT GTC AAG TAG ATT TGC AGG GGG A, 5′-TCA CCA TGC CTG TAA TCC ATA CCC AAA AAA ATA GCA CGC C, 5′-GGA TTA CAG GCA TGG TGA GCA AGG GCG AG, 5′-ATG ACT CGA GCC CGG GTT ACT CGA GGT CTT CTT CGG AAA TCA ACT TCT; Sec12p-sfGFP 5′-CGG ATC CGG CAT CGT CGA TTT CTC TAA AGG TTG CG, 5′-TAG AAC TAG TGG ATC ATG AAG TTC GTT ACA GCA AGC TAC AA, 5′-ACG ATG CCG GAT CCG AAA ACC TGT ACT TCC AG, 5′-AAT TAC ATG ACT CGA TCA TTT GTA GAG CTC ATC CAT GCC ATG T, Bet1p-sfGFP 5′-AGC TGG ATC CAT GAG CAA AGG AGA AGA, 5′-ACG TCT CGA GTC ATT TGT AGA GCT CAT. The sfGFP coding sequence was mutated at position 206. The code for an alanine was exchanged against the code for lysine to prevent dimerization^35^. Vectors were transformed into BY4547 cells kindly provided by Prof. Karin Breunig (Institute of Genetics, University of Halle-Wittenberg, Germany) using the protocol by Akada *et al*.^36^ with the modification that salmon sperm DNA (Thermo Fisher) was used instead of RNA. Sequence of resulting vectors were verified by sequencing.

### Cell culture

Yeast cells were grown in minimal medium with 2% (w/v) raffinose as carbon source at 30 °C, shaking at 120 rpm until an optical density of 0.6 at 600 nm was reached. For gene expression 2% (w/v) galactose were added. Cultures were incubated for 14 h at 20 °C with shaking.

### ER-membrane preparation

ER membranes (microsomes) were purified as described by Wuestehube and Schekman in 1992^21^ with the following modifications: For cell wall digestion, in addition to lyticase, chitinase (0.5 U ml^−1^) was added and the incubation time prolonged to two hours. After the sucrose gradient centrifugation, the microsome fraction was pelleted and resuspended in 19% (v/v) OptiPrep (Sigma-Aldrich), which was part of a nine step OptiPrep gradient with concentrations ranging from 24% to 10% (v/v) OptiPrep. The gradient was centrifuged for 16 h at 4 °C at 100.000 × g (acc5/dec7) in an MLS50 swing out rotor (Optima Max-XP, Beckmann). The membrane fraction with the highest density was harvested, pelleted and resuspended in B88-buffer (20 mM HEPES, pH 6.8, 150 mM potassium acetate, 5 mM magnesium acetate, 250 mM sorbitol) for further use. To confirm the purity of the ER fraction, organelle-specific marker proteins were quantitated by either a western blot or a functional enzyme assay. Western blots were carried out with antibodies against Sec61p as an ER marker (anti-Sec61p kindly provided by Prof. Martin Spiess), against Kex2 for the Golgi (abcam), against Nop9 for the nuclear membrane (Anti-Fibrillarin, Antikoerper-online.de) and against V-ATPase1 for the plasma membrane (Thermo Scientific). Enzyme assays were performed for the vacuolar marker γ-glutamyltransferase (BioVision) and the mitochondrial protein succinate dehydrogenase (Sigma Aldrich). For each marker, the ratio of the signals obtained on the final fraction (i.e., the harvested optiprep fraction) and on the starting fraction (i.e., the spheroplasts) was calculated in order to evaluate the degree of purification of the ER membranes. A marker ratio above one represents enrichment and a marker ratio below one represents depletion of the respective organelle in the final fraction.

### Fusion assay

Microsomes were diluted in B88 buffer to a protein concentration of 0.5 mg ml^−1^ as determined by a Bradford assay and mixed with the membrane dye FM1-43 (4-[2-[4-(dibutylamino)phenyl]ethenyl]-1-[3-(triethylammonio)propyl]pyridinium dibromide, available as MM1-43 from Abcam). The labeled microsomes were divided into 6 aliquots, mixed with 0, 2, 5, 10, 20, or 50 mM guanosine-5′-triphosphate (GTP) and incubated for 2 h. Images at different locations were taken on a Zeiss LSM 710 confocal microscope. For quantitation, GERVs which had a minimum diameter of 2 µm and at least one third of the membrane free of aggregates were taken into account. Data shown are from four different membrane preparations.

### Apyrase assay

After 2 h of GERV formation with 5 mM GTP, 0.01 U ml^−1^ apyrase (based on the specification by Sigma-Aldrich) was added. GERVs were monitored by confocal microscopy after 30 min, 2 h and 4 h.

### Immunostaining procedure

FM1-43 labeled GERVs were grown at 5 mM GTP for 2 h in a 96-well plate with glass coverslip bottom, filled with 150 µl of a microsomal membrane suspension. The supernatant was carefully reduced and replaced by a solution of anti-Sec61p antibody from rabbit (kindly provided by Prof. Martin Spieß) and 3% (w/v) bovine serum albumin (BSA) in B88-buffer (20 mM HEPES, pH 6.8, 150 mM potassium acetate, 5 mM magnesium acetate, 250 mM sorbitol). After 1 h of incubation the sample was washed 3 times with B88-buffer. After each buffer exchange the sample was allowed to rest for 15 min. Afterwards, the secondary antibody (anti-rabbit-AlexaFluor647, from abcam) in B88-buffer with 3% BSA was added and incubated for 1 h. Again, the sample was washed 3 times with B88-buffer.

### Confocal fluorescence microscopy

Membranes were imaged on an LSM710/ConfoCor3 setup (Carl-Zeiss, Germany) using a 40 × /1.2 N.A. water immersion objective. FM1-43 and sfGFP were excited with a 488 nm laser line, mCherry was excited with a 561 nm laser and AlexaFluor647 with a 633 nm laser. A 488/561 or 488/561/633 main beam splitter was used. Two-color images were taken sequentially in different tracks. Fluorescence was detected using photomultiplier (PMT) detectors. For sfGFP and FM1-43, fluorescence was collected in the range of 495 to 595 nm, for mCherry from 585 to 740 nm and for AlexaFluor647 from 655 to 740 nm. Using the Carl-Zeiss ZEN2009 software, a non-linear intensity look-up table with γ = 0.45 was applied in Fig. 1c (right panel only), 3a-c, 3e-f, and 4a-c to facilitate discernibility of membranes.

### Fluorescence correlation spectroscopy (FCS)

Microsomes purified from Sey1p-cells expressing Bet1p-sfGFP and those purified from Sey1p-cells expressing Bet1p-mCherry were mixed at a 1: 4000 ratio and incubated with 5 mM GTP for 2 h at room temperature. The ratio was chosen to ensure that the resulting GERVs have a low density of green-fluorescent Bet1p-sfGFP that is suitable for FCS and a higher density of red-fluorescent Bet1p-mCherry that facilitates visualization of the GERVs by confocal microscopy.

FCS analysis using the LSM710/ConfoCor3 setup was performed on 8 GERVs (from two organelle preparations) and 6 GUVs, which were at least 4.5 µm in diameter. For the diffusion measurements, the membrane at the top of the Bet1p-sfGFP-GERV or Bet1p-Alexa488-GUV (see SI) was placed in the center of the FCS detection volume using confocal imaging, followed by an axial scan of the FCS detection volume to find the maximum count-rate. FCS measurements were performed using a 40 × /1.2 N.A. water immersion objective, the 488 nm argon ion laser line, 5.9 µW laser power for sfGFP and 47 µW for Alexa488 (see SI). A 488/561 main beam splitter and an emission filter of 505–540 nm was used for sfGFP and a 488 main beam splitter and a longpass emission filter (505 nm) for Alexa488. An avalanche photo diode was used for detection.

Measurements were performed for at least two times 30 s. The fluorescence fluctuations were software-correlated and fit with the standard FCS model equation *G(τ)* for 2D-diffusion and fluorophore blinking using the ZEN2009 software,

1 $$G(\tau )=\frac{1}{N}(1+\frac{{F}_{dark}\,{e}^{-\frac{\tau }{{\tau }_{dark}}}}{1-{F}_{dark}})\frac{1}{1+\frac{\tau }{{\tau }_{diff}}}$$

where *N* is the average number of particles, *F*_*dark*_ the average fraction of fluorophores in the dark state, *τ*_*dark*_ the dark state relaxation time and *τ*_*diff*_ the diffusion time.

The resulting diffusion time *τ*_*diff*_ was used to calculate the diffusion coefficient *D,*

2 $$D=\frac{{\omega }_{o}^{2}}{4{\tau }_{diff}}$$

where *ω*_*o*_ represents the lateral 1/e² radius of the detection volume.

To calibrate the size of the detection volume, the diffusion of Alexa488-hydrazide was measured. The resulting *ω*_*o*_ was determined using a diffusion coefficient of Alexa488-hydrazide of 435 µm^2^ s^−1^ ^37^.

## Supplementary information

Supplementary information.
