# Supplementary material for: Giant Endoplasmic Reticulum vesicles (GERVs), a novel model membrane tool
Source: Sci Rep. 2020 Feb 20;10:3100. doi: 10.1038/s41598-020-59700-1 (PMC7033103; doi:10.1038/s41598-020-59700-1)
Supplement: Supplementary file 1 — Supplementary information. [file 41598_2020_59700_MOESM1_ESM.pdf]

Supplementary Information for

Giant Endoplasmic Reticulum vesicles (GERVs), a novel model membrane tool

Mona Grimmer and Kirsten Bacia*

Supporting methods

GTP depletion using Apyrase

Apyrase GTP hydrolysis efficiency was analyzed using HPLC. 10 mM GTP was mixed with 0.01 U ml^{-1} apyrase (based on the specification by Sigma-Aldrich) and incubated for 0 min, 44 min or 84 min. Hydrolysis was traced by separating the nucleotide species by HPLC using a C18 column (Jupiter 5u C18, 300 A, 250 mm x 4.6 mm, 5 µm, Phenomenex). A running buffer containing 50 mM KH_2PO_4, pH 6.3, 10 mM tetra-*n*-butylammonium bromide, 5% (v/v) acetonitril was used isocratically at a flow rate of 0.8 ml min^{-1}.

Bet1p purification and labeling

Bet1p with a cysteine, inserted at the second position of the amino acid sequence, was purified as a GST fusion protein. The pETGEXCT vector with the modified Bet1p sequence was kindly provided by Randy Schekman. After cell disruption via French press in buffer A (25 mM Tris (pH 7.5), 400 mM KCl, 10% (v/v) glycerol, 2% (w/v) *n*-octyl-β-D-glucopyranoside) the protein was loaded onto a GSH-agarose column. After buffer exchange to thrombin cleavage buffer (25 mM Tris (pH 7.5), 250 mM potassium acetate, 5 mM $CaCl_2$, 5 mM $MgCl_2$, 2 % (w/v) *n*-octyl-β-D-glucopyranoside) the GST-tag was cleaved using 1 U ml^{-1} thrombin for 3 h at room temperature and Bet1p was eluted. Bet1p was incubated with an excess of Alexa488-C5 maleimide (Invitrogen). The labeling reaction was incubated for 2 h at room temperature, followed by gel filtration (PD10 column, GE Healthcare) to remove unbound dye.

GUV formation

Bet1p-Alexa488 was incubated with a dried lipid film, prepared from the major-minor-mixture [1,2], in the presence of 2% (w/v) *n*-octyl-β-D-glucopyranoside at a protein to lipid molar ratio of 1 to 1000 for 2 h. Liposome formation was achieved by removing the detergent on a gel filtration (PD10-column, GE Healthcare) with water as mobile phase. Formed liposomes were mixed with sucrose to a final sucrose concentration of 1 M, transferred to a centrifuge tube and overlaid with 900 µl 0.75 M sucrose and 300 µl water. The gradient was centrifuged for 2 h at 264,000 x g (acc5/dec7) at room temperature. The upper vesicle-containing fraction was harvested and concentrated to 100 µl. 2 µl-drops were spotted onto indium tin oxide (ITO) covered glass slides, and dried at 4 °C under vacuum prior to electroformation [3]. The electroformation chamber was built from two ITO slides (Delta Technologies, Stillwater, NM, USA) with the lipid films facing inwards and a 3 mm silicon ring as a spacer. The

chamber was filled with a 500 mOsm kg^{-1} sucrose solution in water. A sinusoidal voltage of 3 V (effective voltage) with a frequency of 10 Hz was applied for 7 h. GUVs were concentrated by adding the suspension of sucrose-containing GUVs to 3 times the volume of 500 mOsm kg^{-1} glucose solution and allowing sedimentation at 4°C.

Quantitative fluorescence image analysis

Angle-dependent fluorescence along the equators (Fig. S4) was evaluated on GERVs exceeding a diameter of 50 pixels [10.4 µm]. To this end, two concentric rings with radii differing by 10 pixels were positioned manually in the confocal image to enclose the equator of the GERV. Using MatLab (MathWorks, Natick, MA), the sum of the fluorescence intensity values of the pixels located between the two rings was calculated with binning to an angle of 1 degree. A moving average over 10 degrees was applied.

Supporting data

Sequence data

Bold printed bases are verified by sequencing.

SEY1 sequence provided by Dharmacon in a BG1805 vector

```
ATGGCTGATAGACCTGCTATTCAGTTGATAGATGAAGAGAAGGAATTTCATCAAAGTGCATTACAATA
TTTTCAACAATGTATTGGAAATCGTGATGTTGGTCTAGATTACCATGTCATCTCAGTTTTCGGTTCTC
AATCGAGTGGTAAATCGACTTTACTTAACGTCTTGTTTAACACCAACTTTGATACCATGGATGCTCAG
GTGAAAAGGCAGCAGACTACTAAAGGCATTTGGTTGGCTCACACTAAACAGGTTAATACAACTATTGA
AATCGATAATGATCGTCCAGATATTTTTGTGCTAGATGTTGAGGGCTCCGATGGTTCAGAAAGAGGTG
AAGACCAAGATTTTGAGAGAAAAGCCGCTTTATTTGCCATTGCAGTATCTGAAGTTCTTATAGTTAAT
ATGTGGGAGCAGCAAATTGGGCTATATCAAGGTAATAATATGGCTTTATTAAAAACAGTTTTTGAGGT
CAACCTTTCCCTATTTGGCAAGAATGATAATGACCACAAGGTGCTGTTACTTTTTGTAATTAGAGATC
ATGTAGGTGTGACTCCACTCTCGAGTTTGAGTGATTCCGTTACGAGAGAATTGGAAAAAATATGGACA
GAGTTAAGTAAACCTGCTGGTTGTGAGGGTTCTAGCCTGTATGATTATTTCGATTTAAAGTTTGTCGG
GCTAGCTCATAAGTTGTTGCAAGAAGACAAATTTACCCAGGATGTCAAGAAATTAGGTGATTCGTTTG
TGATGAAAGGTACGGAAAACTATTATTTCAAACCTCAATATCACCATAGGTTGCCATTAGATGGTTGG
ACTATGTACGCAGAAAATTGTTGGGATCAAATTGAACGTAACAAGGATTTAGATCTTCCAACTCAGCA
AATTCTGGTTGCTAGATTTAAAACTGAAGAAATATCTAATGAAGCTCTAGAAGAGTTCATTTCAAAAT
ATGATGAATCGATTGCTCCTTTAAAGGGTAACTTGGGATCTTTAACATCTCAGCTAGTGAAGCTAAAA
GAAGAATGTCTGACAAAATATGATGAACAAGCATCGCGCTATGCAAGAAACGTTTATATGGAGAAACG
AGAAGCTTTAAATACAAAGCTGAATTCACATATTTCAGGTACAATTAATGAGTTCTTGGAATCATTAA
TGGAAAAGTTATGGGACGATTTAAAATTGGAGGTGTCTTCTAGAGACAAAGCTACTACCTCTTTTGTA
GAAAGTGTGGCGGCAGGTAAGAGTAAGATTGAGAAAGAATTTAACGAATCAATGGAGACCTTCAAAAA
GTTAGGGCTACTAATATCAAACGAAGAGATTACCTGCAAATTTTCCGACGATATTGAAGAAAGAATCA
AGCAACTACGTGATGCTGAATTGAAAGCAAAAATCGGTCGTATTAAAAAAAATCTGGTTCCCGAATTA
AAGGATCATGTGATTCATTTACTATCACATCCATCCAAAAAGGTTTGGGATGATATAATGAACGATTT
TGAATCTACTATCAAAGACAATATATCTGCGTATCAAGTGGAAAAAGATAAATATGATTTTAAAATTG
```

GACTTTCAGAGAGCGAAAACGCGAAGATTTACAAAAATATTAGAATA**TTGGCGTGGAGAACCTTGGAT**
ACCACAGTACATGACTACCTGAAAATAGATACGATTGTTAGTATATTGAGAGACAGGTTTGAAGATGT
ATTCAGATATGATGCTGAAGGTTCTCCAAGATTGTGGAAAACAGAAGAAGAGATTGATGGGGCATTCC
GTGTTGCAAAAGAACATGCCCTGGAAGTTTTTGAGGTTCTTTCACTTGCTGTAACCTCAGATAACGTT
GAAATTATTCCTGATGTACCAATGGCTGAAGAGGAAAGCGGCGAGGACAACGAAATATATCGGGACAA
CGAAGGTGTGTTTCATTCCCGTCGTTTCGCACACATCTTGACTGAATTGCAAAAGGAGAATGTTTTAG
ATCAATTCCGCAGGCAGATTAACATTACTGTTCTGGATTCGAAAAGGTCTATCATCACTACTAGAACG
CATATTCCACCTTGGATTTACGTTTTGCTAGCCGTATTAGGGTGGAACGAATTCGTGGCTGTCATAAG
AAACCCCTTATTTGTAACCCTTACCTTGATCTTAGGTGCAACCTTTTTTGTTATTCATAAGTTCGGCC
TCTGGGGCCCTGTTGTTAATGTTGTTCAAAGTGCAGTTGGTGAAACAAGAACTGCAATAAAGGATAAA
CTAAGGCAATTTGTCGTTGAAGATCATGAAGTGAAGGAATCTTTTGAAATGAAAGATTTCTCAAAAAA
CGAGCAAAAAGAAAAATGA

BET1 sequence

ATGTCAAGTAGATTTGCAGGGGGAAACGCTTATCAACGTGATACTGGTAGAACACAGTTATTCGGACC
GGCTGATGGATCAAATAGTCTCGATGACAATGTATCATCAGCGCTAGGGAGCACAGATAAATTAGACT
ACTCCCAAAGTACTTTGGCATCTCTTGAATCTCAAAGTGAGGAACAGATGGGAGCTATGGGTCAGAGA
ATAAAAGCACTCAAGTCATTATCGTTGAAGATGGGTGATGAGATTAGAGGCAGCAATCAAACTATTGA
CCAGCTTGGTGATACTTTCCATAACACTTCTGTAAAACTCAAAAGGACTTTTGGAAACATGATGGAGA
TGGCCAGAAGATCTGGGATCAGTATAAAAACATGGTTAATAATATTTTTTATGGTAGGCGTGCTATTT
TTTTGGGT___ATGGATTACAGGCATGTGAGCAAGGGCGAGGAGGATAACATGGCCATCATCAAAGGAGTT___
CATGCGCTTCAAGGTGCACATGGAGGGCTCCGTGAACGGCCACGAGTTCGAGATCGAGGGCGAGGGCG
AGGGCCGCCCCTACGAGGGCACCCAGACCGCCAAGCTGAAGGTGACCAAGGGTGGCCCCCTGCCCTTC
GCCTGGGACATCCTGTCCCCTCAGTTCATGTACGGCTCCAAGGCCTACGTGAAGCACCCCGCCGACAT
CCCCGACTACTTGAAGCTGTCCTTCCCCGAGGGCTTCAAGTGGGAGCGCGTGATGAACTTCGAGGACG
GCGGCGTGGTGACCGTGACCCAGGACTCCTCCCTGCAGGACGGCGAGTTCATCTACAAGGTGAAGCTG
CGCGGCACCAACTTCCCCTCCGACGGCCCCGTAATGCAGAAGAAGACCATGGGCTGGGAGGCCTCCTC
CGAGCGGATGTACCCCGAGGACGGCGCCCTGAAGGGCGAGATCAAGCAGAGGCTGAAGCTGAAGGACG
GCGGCCACTACGACGCTGAGGTCAAGACCACCTACAAGGCCAAGAAGCCCGTGCAGCTGCCCGGCGCC
TACAACGTCAACATCAAGTTGGACATCACCTCCCACAACGAGGACTACACCATCGTGGAACAGTACGA
ACGCGCCGAGGGCCGCCACTCCACCGGCGGCATGGACGAGCTGTACAAGATGGAACAGAAGTTGATTT
CCGAAGAAGACCTCGAGTAACC

Bet1p-coding sequence is underlined and mCherry-coding sequence is written in italics.

Sec12p-sfGFP sequence

ATGAAGTTCGTTACAGCAAGCTACAATGTGGGCTACCCTGCCTATGGCGCAAAATTCCTGAACAATGA
CACTTTACTGGTGGCGGGCGGTGGCGGTGAAGGCAATAACGGTATTCCTAACAAATTAACCGTTTTGC
GTGTTGATCCAACAAAAGACACGGAAAAGGAACAATTTCATATCCTGAGCGAATTTGCCTTAGAAGAT
AACGATGACAGTCCTACTGCCATTGATGCGTCCAAAGGCATTATCCTGGTGGGTTGTAATGAAAACAG
TACCAAGATTACTCAAGGCAAAGGTAATAAGCACTTAAGAAAATTCAAATACGATAAGGTAAACGACC
AGCTGGAATTTTTAACAAGTGTTGATTTCGACGCCTCCACCAATGCGGATGACTATACTAAACTGGTC
TACATTTCACGCGAAGGTACTGTAGCTGCAATTGCCTCTTCAAAAGTTCCAGCGATCATGCGCATTAT
CGATCCGTCGGACTTAACAGAAAAGTTCGAAATCGAAACGCGTGGCGAAGTTAAAGATTTGCATTTCT
CAACAGACGGCAAGGTTGTGGCTTACATTACGGGTAGCAGTCTGGAAGTTATCTCAACAGTGACGGGT

AGCTGCATCGCTAGAAAGACAGATTTCGACAAGAACTGGAGCCTGAGTAAAATTAACTTCATCGCAGA
TGACACGGTGTTGATCGCCGCGTCCCTGAAAAAGGGCAAAGGTATTGTCTTGACCAAAATTAGCATCA
AGAGTGGTAATACTAGTGTTCTGCGTTCCAAACAAGTGACCAACCGTTTTAAAGGCATTACTTCTATG
GATGTGGACATGAAGGGTGAATTGGCTGTCCTGGCATCGAATGATAACTCTATCGCTTTAGTGAAATT
GAAGGATCTGTCCATGTCGAAAATTTTTAAGCAGGCTCATTCTTTCGCAATTACAGAAGTTACGATCT
CCCCGGATTCGACATATGTCGCCTCTGTATCAGCTGCAAATACGATCCACATTATCAAATTGCCTCTG
AACTACGCGAACTACACCTCGATGAAACAAAAGATCTCTAAGTTTTT**CACTAACTTCATCCTGATCGT**
TCTGTTGTCATACATCTTGCAGTTCAGCTACAAGCATAACTTACACTCAATGTTGTTTAATTACGCAA
AGGATAACTTCCTGACCAAGCGCGATACTATCTCCTCGCCATACGTCGTAGATGAAGACTTACATCAG
ACCACTTTGTTTGGCAATCACGGTACCAAAACTTCTGTTCCGTCAGTGGATAGCATTAAGGTCCATGG
CGTACACGAAACCTCTTCAGTCAACGGTACAGAAGTACTGTGTACGGAAAGCAATATTATCAACACTG
GCGGTGCTGAATTTGAAATCACGAACGCAACCTTTAGAGAAATCGACGATGCCGGATCCGAAAACCTG
TACTTCCAGGGTCAATTCAG*CAAAGGAGAAGAACTTTTCACTGGAGTTGTCCCAATTCTTGTTGAATT*
AGATGGTGATGTTAATGGGCACAAATTTTCTGTCAGTGGAGAGGGTGAAGGTGATGCTACATACGGAA
AACTCACCCTTAAATTTATTTGCACTACTGGAAAACTACCTGTTCCATGGCCAACACTTGTCACTACT
CTGACCTATGGTGTTCAATGCTTTTCCCGTTATCCGGATCACATGAAACGGCATGACTTTTTCAAGAG
TGCCATGCCCGAAGGTTATGTACAGGAACGCACTATATCTTTCAAAGATGACGGGAACTACAAGACGC
GTGCTGAAGTCAAGTTTGAAGGTGATACCCTTGTTAATCGTATCGAGTTAAAGGGTATTGATTTTAAA
GAAGATGGAAACATTCTCGGACACAAACTAGAGTACAACTATAACTCACACAATGTATACATCACGGC
AGACAAACAAAAGAATGGAATCAAAGCTAACTTCAAAATTCGCCACAACATTGAAGATGGTTCCGTTC
AACTAGCAGACCATTATCAACAAAATACTCCAATTGGCGATGGCCCTGTCCTTTTACCAGACAACCAT
TACCTGTCGACACAATCTAAGCTTTCGAAAGATCCCAACGAAAAGCGTGACCACATGGGCCCTTCCTG
A

Sec12-coding sequence is underlined and sfGFP_A206K is written in italics.

ER preparation quality

To evaluate the quality of the purification procedure, western blots and functional enzyme assays for specific organelle marker were performed on the final fraction (optiprep) and, for comparison, on the initial material (spheroplasts). The ratio of the signals is shown in Fig. 1b.

To allow the quantitative assessment, sample volumes were adjusted to equal total amounts of protein, applied onto an SDS-gel and transferred to a nitrocellulose membrane for the immune reaction and detection via chemiluminescence. The signal intensities of the bands were quantitated using the ImageJ software. The intensity of the final optiprep fraction was divided by the intensity of the spheroplast fraction. To test for vacuolar membrane and mitochondrial membrane, the enzyme activities measured in the final optiprep fraction were divided by the corresponding activities measured in the spheroplast fraction. Any value smaller than one signifies a depletion of the organelle compared to the starting material and any value larger than one an enrichment (Fig. 1b). We conclude that only the ER membranes (microsomes) are enriched and all other organelle membranes are depleted.

To monitor the role of the different steps of the preparation procedure, further samples were taken and analyzed for organelle markers. The blots for Sec61p and Nop9p (Fig. S1b,c) show how ER and nuclear membranes are separated in the procedure.

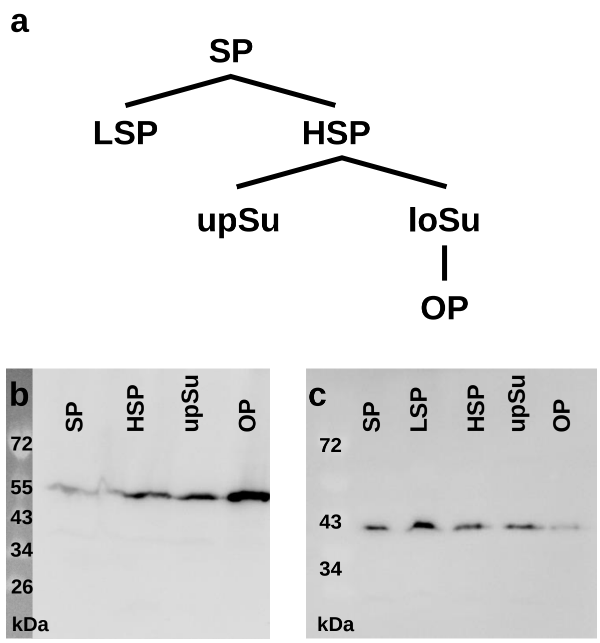

Figure S1. Analysis of ER-membrane preparation procedure (shown schematically in (a)), via western blots and activity assays of organelle markers. Samples from different purification steps were analyzed. A western blot against the ER-resident translocon Sec61p is shown in (b) and one against the nuclear pore protein Nop9 in (c).

SP – spheroplast fraction, LSP – low speed pellet, HSP – high speed pellet, upSu – upper fraction of sucrose gradient, loSu – lower fraction of sucrose gradient, OP – optiprep fraction.

GTP hydrolysis by apyrase

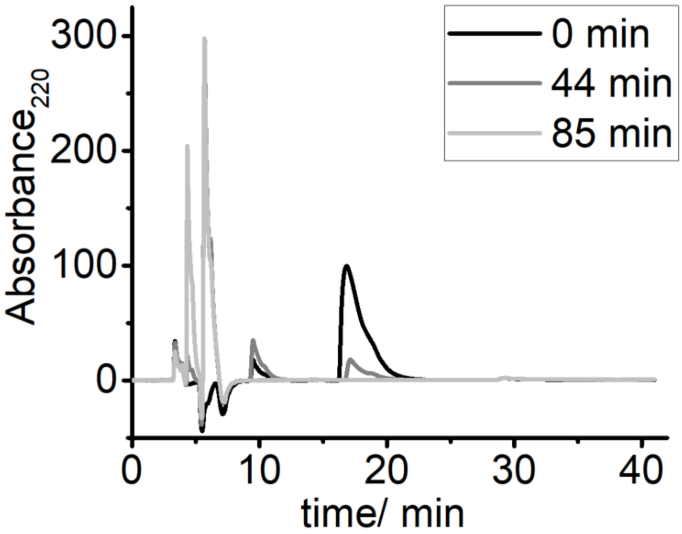

Figure S2. HPLC assay of GTP hydrolysis using 10 mM GTP, mixed with 0.01 U ml^{-1} apyrase, monitored before addition of apyrase (0 min) and 44 and 85 min after addition of apyrase. GTP elutes at 17 ml, GDP at 11 ml.

Diffusion measurement of Bet1p-Alexa488 in synthetic GUVs using FCS

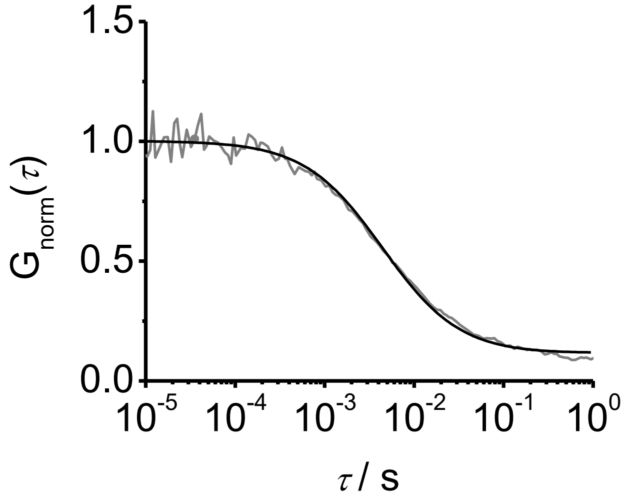

Figure S3. Representative example of an amplitude-normalized fluorescence autocorrelation curve of Bet1p-Alexa488 diffusion in a synthetic GUV lipid bilayer. The FCS detection volume was positioned on the membrane at the top of a GUV.

Measurement of 6 GUVs yielded a diffusion coefficient of (2.4 ± 0.8) $\mu m^2 s^{-1}$. The diffusion of Bet1p in the GUV membrane is ~ 9 fold faster than in GERVs. The effect of the differently sized labels sfGFP and Alexa488, which are linked to the domains located in the aqueous phase, cannot account for this large difference, because the protein's membrane domain, which is located in the phase with the much higher viscosity is the primary determinant of the diffusion [4]. Similar as in the comparison of GPMVs with GUVs, the complex, protein-containing GERV membrane is expected to slow down the diffusion of the Bet1p membrane protein in comparison to a simple, liquid-disordered phase lipid bilayer.

Quality check of GERV membranes by quantitative fluorescence image analysis

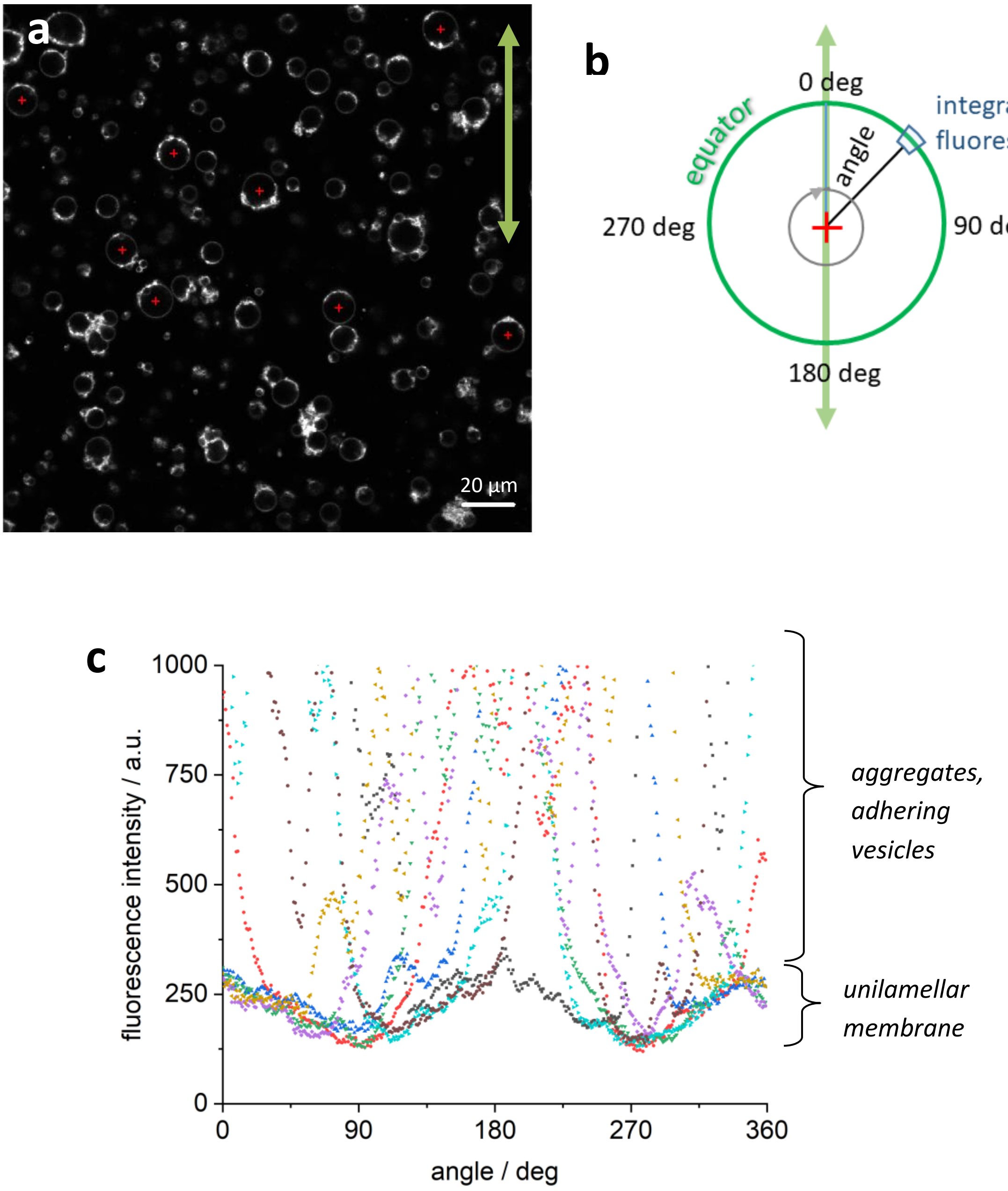

Figure S4. (a) Larger confocal scanning field of view, comprising the image in Fig. 1d. GERVs are fluorescently labeled with the styryl dye FM1-43. The green arrow indicates the polarization orientation of the laser excitation. The red crosses denote the centers of the largest GERVs selected for analysis. (b) Quantitation scheme. (c) Fluorescence intensity as a function of the angle along the GERV equator. Each symbol color denotes a different GERV.

The image in Fig. S4a was focused approximately at the equators of the similarly sized, largest GERVs. To evaluate the intensity of the fluorescence and its angle-dependence along the equators, GERVs with diameters exceeding 50 pixels (10.4 µm), having circular cross-sections and no close apposition to

neighboring GERVs, were selected for quantitative analysis. The centers of the selected GERVs are marked with a red cross. The fluorescence intensity as a function of the angle with respect to the polarization direction of the laser was obtained by integrating pixel fluorescence (Fig. S4b and Supplementary Methods). Fig. S4c shows that there is a unique, but angle-dependent fluorescence intensity, which is shared by all the GERVs. This unique fluorescence indicates a unilamellar membrane, because multilamellar membranes are expected to show integer multiples of this fluorescence intensity.

The restricted orientational freedom of the styryl dye in the lipid bilayer produces a typical angle-dependence [5,6]. Vesicle aggregation that is also obvious from the image (Fig. S4a) by visual inspection causes the spurious excursions to high fluorescence intensities.

References

1 Matsuoka, K. *et al.* COPII-coated vesicle formation reconstituted with purified coat proteins and chemically defined liposomes. *Cell* **93**, 263-275 (1998).

2 Miller, E., Antonny, B., Hamamoto, S. & Schekman, R. Cargo selection into COPII vesicles is driven by the Sec24p subunit. *EMBO J* **21**, 6105-6113 (2002).

3 Daum, S. *et al.* Insights from reconstitution reactions of COPII vesicle formation using pure components and low mechanical perturbation. *Biol Chem* **395**, 801-812, doi:10.1515/hsz-2014-0117 (2014).

4 Saffman, P. G. & Delbruck, M. Brownian motion in biological membranes. *Proc Natl Acad Sci U S A* **72**, 3111-3113 (1975).

5 Demchenko, A. P., Mely, Y., Duportail, G. & Klymchenko, A. S. Monitoring biophysical properties of lipid membranes by environment-sensitive fluorescent probes. *Biophys J* **96**, 3461-3470, doi:10.1016/j.bpj.2009.02.012 (2009).

6 Werner, S. *et al.* Dendritic domains with hexagonal symmetry formed by x-shaped bolapolyphiles in lipid membranes. *Chemistry* **21**, 8840-8850, doi:10.1002/chem.201405994 (2015).
